# MEMSbased Double-Stacked Tower Biosensor Array with Integrated Readout Circuitry for Detection of Salivary pH as a Diagnostic Biomarker Applied for Chronic Periodontal Disease

**DOI:** 10.3390/s22228652

**Published:** 2022-11-09

**Authors:** Wei-Cheng Lin

**Affiliations:** 1 Department of Electrical Engineering, Chang Gung University, No. 259, Wenhua 1st Rd., Guishan Dist., Taoyuan 33302, Taiwan; weiclin@mail.cgu.edu.tw; Tel.: +886-3-211-8800 (ext. 3221); Fax: +886-211-8026; 2 Department of Trauma and Emergency, Linkou Chang Gung Memorial Hospital, Guishan Dist., Taoyuan 33305, Taiwan

**Keywords:** pH, saliva, MEMS, readout, SoC

## Abstract

MEMS based 3D double stacked tower pixel biosensor 10 × 10 array with integration of readout circuit for detection of saliva pH ion is demonstrated. The pixel biosensor comprised a driving electrode, sensing electrode and double stack tower pixel structure. The sensitivity of double stacked tower biosensor can be auxiliary enhanced by proposed lower-jitter low dropout regulator circuit and dual offset cancellation comparator. The double stacked tower sensor is fabricated by MEMS backend-of-line CMOS process, it is compatible with CMOS frontend readout circuits and integrated as a system-on-chip (SoC). The double stacked tower pixel by MEMS process is to obtain a larger volume ratio of charge groups in a pixel of biosensor to enhance the sensitivity and linearity for ion detection. With the double stacked tower structure in biosensor, the sensitivity is improved by 31% than that of single tower structure proved by simulation. A wide-range linearity from pH 2.0 to pH 8.3, high sensitivity of −21 ADC counts/pH (or 212 mV/pH), response time of 5 s, repetition of 98.9%, and drift over time of 0.5 mV are achieved. Furthermore, the proposed biosensor was performed to confirm the artificial saliva from healthy gingiva, chronic gingivitis and chronic periodontitis, the measured ADC counts from proposed biosensor SoC was in consistent of that measured cyclic voltametric (CV) method very well. The proposed 3D double stack tower biosensor and readout circuit can be further integrated with internet-of-thing (IoT) device and NFC for data transmission for continuous pH sensing to facilitate the chronic gingiva disease health care at home.

## 1. Introduction

Oral diseases such as periodontitis and oral malodor are always initiated at the interface between microbial ecosystem and gingiva tissue. The changes in microbial in the microbial systems increase the risk for pathogenicity and promote oral disease. It is well known to use clinical parameters including probing depth, attachment level, bleeding on probing plaque index, or radiographic loss of alveolar bone to assess health of an oral gingiva, however these methods are risk for oral disease patients and a challenge for both clinicians. There are strong evidences [1,2] associating the effect of the pH on the growth of microorganisms, for example *P. gingivalis* grows at pH of 6.5–7.0, *P. intermedia* grows at 5.0–7.0, and *F. nucleatum* grows at pH 5.5–7.0. Therefore, there is a compelling reason that oral saliva can be used as a diagnostic biomarker [3,4].

Using the saliva as a diagnostic biomarker meets the demand for being inexpensive, non-invasive, and easy to use for patients. As a clinical tool, saliva has many advantages over serum, it is easy of collection, storing, and can be obtained at very low cost. For the patients, the benefit of non-invasive method reduces the patients’ anxiety and discomfort. Meantime, Saliva is easier to handle for diagnostic procedures because it does not clot, thus reducing the manipulations. The pH baseline is within a narrow range of pH 7.0–7.5 for a healthy person [5]. Even pH levels drop below 5 after drinking an acidic substance, it reverts back to the baseline value after one hour. A lasting low pH episodes stimulate bacterial growth in the oral. Thus, saliva shows a major influence on plaque initiation, maturation and metabolism, and can identify health conditions such as diabetes, inflammation, infection and hormonal perturbations. Therefore, the detection of saliva pH level plays a diagnostic marker in periodontal disease.

In the past, there were lot of papers investigating the ion sensors for biomedical field, such as potentiometric sensors [6], conductimetric sensors [7], ion sensitive field effective transistors (ISFET) sensors [8], and extended gate field effect transistor (EGFET) sensors [9]. Among them, ISFETs are most investigated owing to their compatibility with integration circuit and miniaturization. For the sensing configuration of the ISFET, gate terminal is replaced by electrolyte solution, a reference electrode and sensing material that determine the sensitivity. When ISFET is immersed in the solution, a gate terminal is filled with ions, thereby generating a current change between the source and drain terminal of the ISFET. The more the ions filled in the gate terminal of ISFET, the greater the conduction current in ISFET channel. The ion numbers in range pH 5.0–8.0 was less than that in pH 2.0–5.0, therefore the poor sensitivity and nonlinearity were restricted in the range pH 2.0–8.0. In [10], it demonstrated a sensitivity of 140 mV/pH with narrow-range linearity of pH 6–7.6; ref. [11] verified the sensing film and showed a sensitivity of 49.63 mV/pH with base pH level 7–12, the base was for industrial sensing but not suitable for biomedical applications. [12,13] redesigned the reference electrode with palladium oxide, it exhibited a wide-range linearity pH 2–12 detection but no curve was shown, and no application matrix was published, with a moderate sensitivity of 57 mV/pH–62 mV/pH. The article did not analyze other parameter since it focused on the sensor’s electrical apparatus. The linearity of ion sensor is defined as the activity ratio of upper and lower detection limit and approximately corresponds to the range where the electrode responds according to the Nernst equation. All of the above papers only emphasize the sensitivity and work in a narrow pH range, lacking linearity analysis and improvement over a wide pH working range.

In this regard, we propose a 3D double stacked tower biosensor structure to achieve a wide-range linearity for pH 2.0–8.0 and higher sensitivity. The 3D double stacked biosensor structure in a unit pixel comprised a driving electrode, sensing electrode, and double stack towers, the towers in pixel increases the volume ratio of the unit pixel in sensor, which provides a relatively strong internal capacitive coupling effect to improve the sensitivity and linearity for low ions in pH 5.0–8.0 condition. Moreover, the 3D biosensor structure is fabricated by back-end of line MEMS process, it is compatible with CMOS front-end process. Therefore, the 3D double stacked tower biosensor is integrated with circuit of low-jitter low drop regulator and dual offset cancellation technique in comparator. Owing to the increase of the volume ratio for sensor surface area and implementation of readout circuit, the proposed biosensor exhibited wide-range linearity of pH 2.0 to pH 8.3, high linearity of −21 ADC counts/pH (or 212 mV/pH), response time of 5 s, repetition of 98.9%, and drift over time of 0.5 mV. Finally, the proposed biosensor was performed to confirm the artificial saliva from healthy gingiva, chronic gingivitis and chronic periodontitis, the measured ADC counts were in consistent of that measured cyclic voltametric (CV) method very well.

## 2. Materials and Methods

### 2.1. Double Stacked Tower Biosensor Pixel Design and MEMS Fabrication Flow

#### 2.1.1. Proposed Pixel Structure

**Figure 1:**
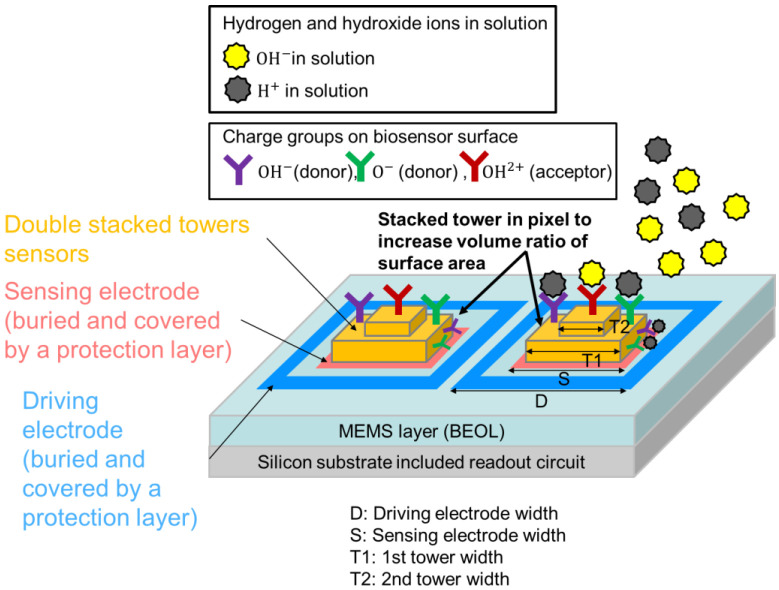
Cross-sectional view of the proposed 3D stacked tower pixels in biosensor array before and after specific ions binding. Charge groups are on the tower surface, and ions hydrogen and hydroxide are in analyte solution.

Sensitivity is very important for ion biosensor, we expect the maximum output change in electrical field in a unit pixel of biosensor. There are lots of papers studying the optimization for biosensor and its sensing principle, such as Donnan boundary potential model [11], ion exchange theory [12], oxygen interaction [13], and site-binding theory [14,15]. In those papers, site-binding theory has been investigated actively for calculating the surface charge for the potential difference. Based on the site-biding theory, as shown in Figure 1, the charges of O^−^, OH^−^ and OH^2+^ are formed on the surface biosensor when biosensor immersed in the analyte solution. The charges on biosensor surface of stack towers are treated as acceptors and donors, they will react with hydrogen and hydroxide ions and attract the ions in solution. The larger the surface area, that is, the larger the volume ratio of the biosensor surface, the higher the sensitivity.

Figure 1 shows the proposed 3D double stacked biosensor pixel array. The sensor array is fabricated by the micro electric mechanical systems (MEMS) process after the silicon frontend process. For each pixel in sensor array, the pattern of driving electrode (D) and sensing electrode (S) is shown in Figure 1, the clear distance between driving electrode and sensing electrode determines the electrical filed strength to detect ion binding. A double stacked tower is fabricated on sensing electrode, the optimization of the 1st tower width (T1) and 2nd tower width (T2) increases the charges group of OH^−^, O^−^ and OH^2+^, the more charges group on the tacked tower, the higher the sensitivity of the electrical field strength due to ion binding.

#### 2.1.2. The Operational Principle of Electrical Field Change for Ion Binding

**Figure 2:**
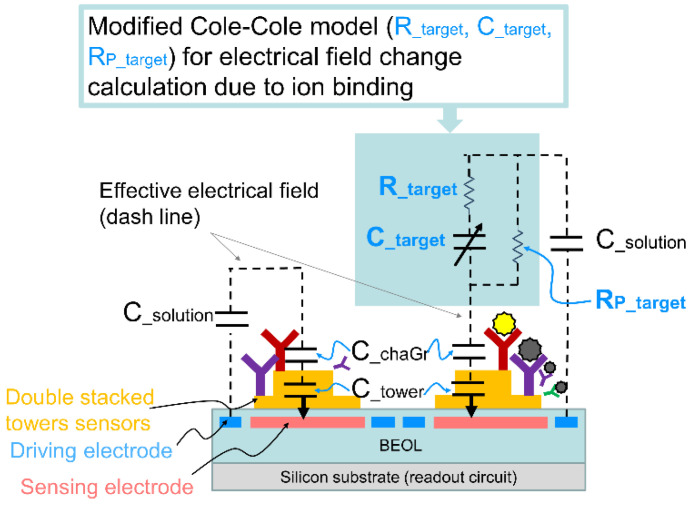
The operation principle of electrical field change when ion binding occurs based on the modified Cole-Cole model. The interaction between charge groups on stacked tower and ions in solution result in the electrical field change. These variations can be sensed as a capacitance change between the driving electrode and stacked tower on the sensing electrode. The saturation point happens on the pH value above pH 10 and below pH 3, in which pH range the modified Cole-Cole model shown in Figure 2 can’t be applied due to high concentration of ions leading a strong electrical field.

Figure 2 depicts the electrical field effective model for the proposed 3D double stacked tower biosensor array with the ions binding, which is modified from the Cole-Cole model [16,17]. The charge groups OH^−^, O^−^ and OH^2+^ gather on the stacked tower. When there are no charges on the stacked tower, the effective electrical field model can be described as a C__solution_ in series a C__chaGr_ and a C__tower_. C__solution_ is parasitic capacitance in solution, C__chaGr_ is parasitic capacitance of charge group on stacked tower, and C__tower_ is parasitic capacitance in stacked tower.

When the solution contains the ions, the effective electrical field for the ion binding can be described a R__target_ in series a C__target_, and parallel a R_P_target_, and the binding electrical field model in series with C__chaGr_ and C__tower_ and C__solution_. The R__target_ and C__target_ and R_P_target_ means the electrical impedance of ion binding, which is used to represent the ion binding resistance affected by an object to current which has both frequency independent and dependent behavior. When the measurements are made using AC signals of different frequency, we can identify the properties of ion binding strength.

In order to validate the effective electrical field model, we describe the model total impedance and will validate in different solutions in Equation (1). C__chaGr_, C__tower_ and C__solution_ are inherent parameters, in a frequency domain it is neglect small, we can focus on the model parameter R__target_, C__target_ and C__solution_ for ion binding. In Equation (1), capacitance C__target_ is a constant phase element, we use a fitting parameter α as a dispersion coefficient, which identify the distribution of relaxation time constants within the ion binding behavior and can be related to the ratio of volume of stacked tower sensor.

(1) $$Z=\frac{R_{{}_{target}}\times R_{P_{target}}+R_{P_{target}}\times \frac{1}{jw^{\alpha }C_{{}_{target}}}}{R_{{}_{target}}+R_{P_{target}}+\frac{1}{jw^{\alpha }C_{{}_{target}}}}\;=\frac{R_{P_{target}}+w^{2}C^{2}\left(R_{{{}_{}}_{target}}\times R_{P_{}{}_{target}}\right)\left(R_{P_{}{}_{target}}+R_{{{}_{}}_{target}}\right)}{1-w^{2}C^{2}{\left(R_{P_{{{}_{}}_{target}}+}R_{{{}_{}}_{target}}\right)}^{2}}\;-\;j\frac{wC\times R_{P_{{}_{target}}}}{1-w^{2}C^{2}{\left(R_{P_{{}_{target}}+}R_{{}_{target}}\right)}^{2}}\;=Re\left(Z\right)+Im\left(Z\right)$$

**Figure 3:**
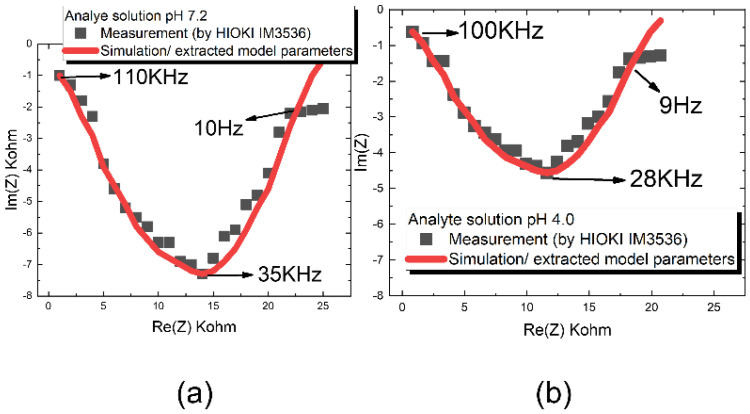
The modified Cole-Cole model using the extracted parameters in Table 1 for (**a**) analyte solution from pH 7.2 and (**b**) analyte solution from pH 4.0 in respectively. The model predicts a well agreement with commercial impedance measurement instrument HIOKI IM3536.

*Re*(*Z*) and *Im*(*Z*) are the real part and imaginary part of the impedance respectively. In order to extract the impedance spectroscopy, we use a periodic triangular-wave voltage as a fixed frequency, it converts to a current by implementing a commercial chipset AD844 current feedback amplifier, and then injected into the effective electrical model. We use two analyte buffers, pH 7.2 and pH 4.0, to simulate the ion binding phenomenon in the stacked tower biosensor array. The frequency of the triangle-wave is selected at an optimized value so long as this value lies within the bandwidth where the dispersion model is applicable. In Figure 3, we measured the analyte buffer pH 7.2 and pH 4.0 to construct the effective electrical field model parameters shown in Figure 2. The periodic triangular-wave frequency runs from 5 Hz to 120 kHz, as shown in Figure 3a,b. Excellent agreement is seen when we compare frequency domain impedance measurement performed by HIOKI IM3536 with the effective electrical field model using the extracted parameter shown in Table 1 for analyte solution pH 7.2 and pH 4.0. The excellent agreement can be seen except for very low frequency of 10 Hz in Figure 3a and 9 Hz in Figure 3b where the model is not applicable.

#### 2.1.3. Fabrication Flow of Stacked Tower Biosensor Array

**Figure 4:**
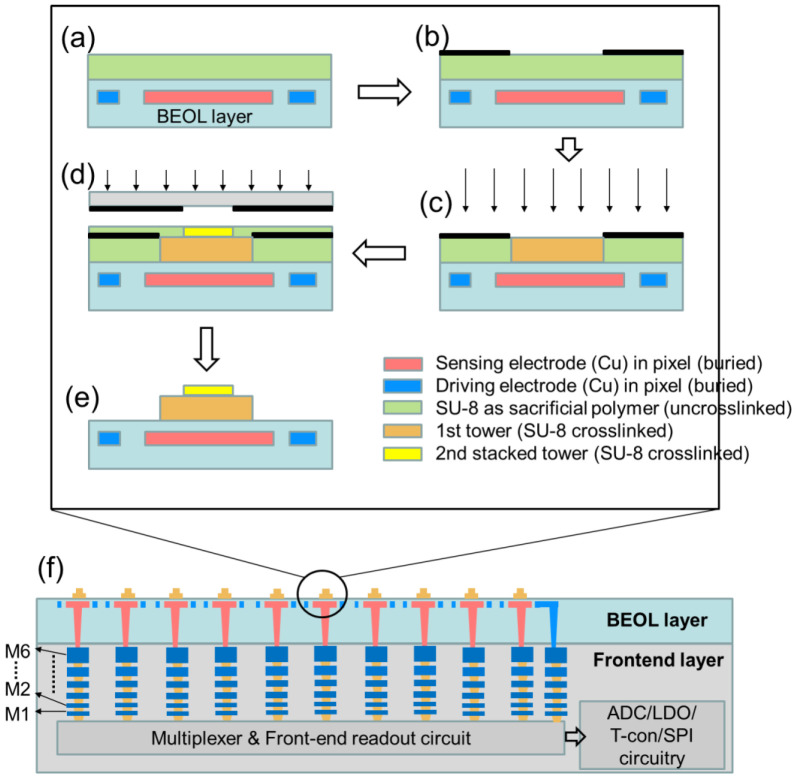
The process flow of proposed MEMS-based double stacked tower biosensor array. (**a**) spin coating of SU-8 as a sacrificial layer, (**b**) deposition and patterning an UV blocking layer, (**c**) blanket exposure to UV, (**d**) spin coating SU-8 and then exposure to UC, (**e**) development and etching the UV blocking layer. (**f**) cross view of integration of MEMS biosensor and front-end readout circuit and signal processing circuit.

Figure 4 illustrates the fabrication flow for the proposed 3D double stacked tower biosensor array the MEMS-based stacked tower sensor array are based on the back-end-of-line process, which is fabricated after the front-end circuit implementation process is being completed. SU-8 is used for micromachining in the proposed stacked tower sensor [18]. SU-8 is a commonly used epoxy-based negative photoresist. In Figure 4a, a SU-8 is spin coated on the back-end-of line (BEOL) layer, the SU-8 is the sacrificial layer, which is uncrosslinked. In Figure 4b, we deposit and pattern an UV-blocking layer, after the UV expositing in Figure 4c, the exposure region is patterned, and 1st tower is crosslinked. In Figure 4d, we spin coat the top layer and again exposure to UV through a mask, the 2nd stacked tower is crosslinked. Finally in Figure 4e, after the development and etching the UV-blocking layer, the proposed double stacked tower biosensor array is constructed. Figure 4f shows the cross- sectional view of the proposed MEMS-based biosensor array on the BEOL layer, which integrates the front-end CMOS process, including the multiplexer circuit, frontend readout circuit, analog to digital convertor as a system-on-chip (SoC).

#### 2.1.4. Pixel Layout and Sensitivity Evaluation of Double Stacked Tower Biosensor

**Figure 5:**
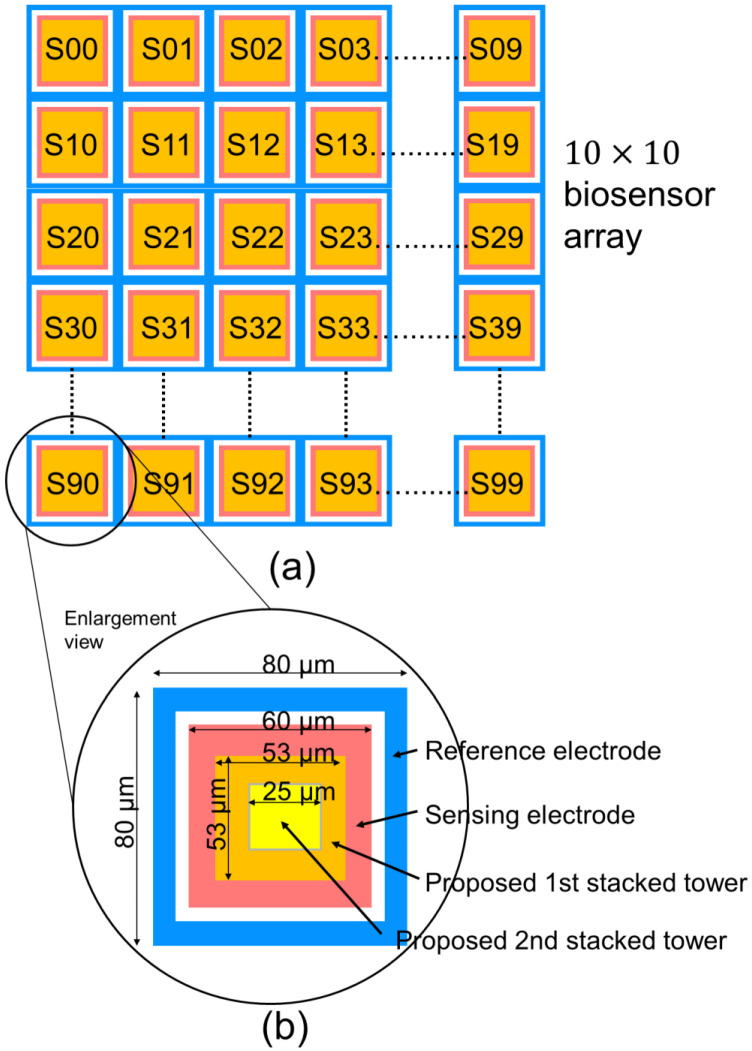
The proposed (**a**) MEMSbased 3D stacked tower 10 × 10 biosensor array and (**b**) geometry dimensions.

**Figure 6:**
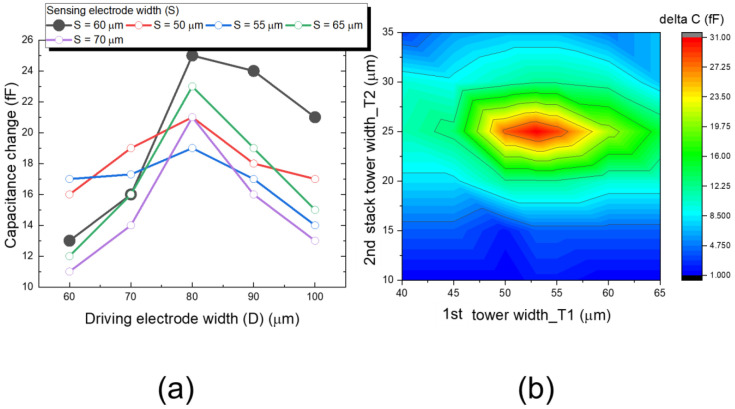
The investigation of (**a**) capacitance change in a pixel geometry dimension for driving electrode width (D) against sensing electrode width (S), and (**b**) contour field plot for 1st stacked tower width (T1) against 2nd stacked tower width (T2).

**Figure 7:**
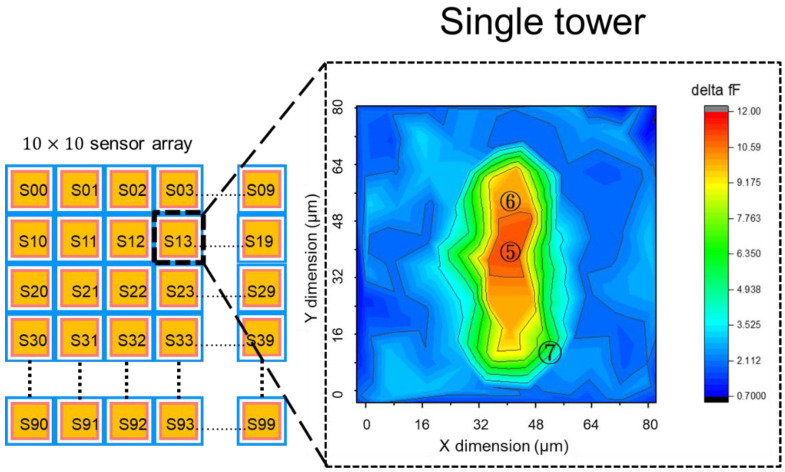
Capacitance change contour plot for single tower in a pixel. The contour plot represents the capacitance change absorbed by ion-binding. The area noted in ⑤_RED is most sensitivity zone (hot zone), and noted in ⑥_YELLOW is the second sensitivity zone and ⑦ is thirdly. The simulation is performed by HFSS. The capacitance change area ⑤_RED, ⑥_YELLOW, ⑦_GREEN account for 36% of a pixel.

**Figure 8:**
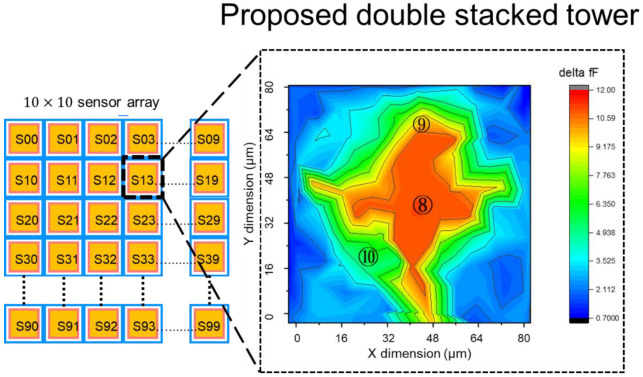
Capacitance change contour plot for proposed doubled stack tower in a pixel. The contour plot represents the capacitance change absorbed by ion-binding. The area noted in ⑧_RED is most sensitivity zone (hot zone), and noted in ⑨_YELLOW and ⑩_GREEN are secondly and thirdly, in respectively. The capacitance change in ⑧_RED, ⑨_YELLOW, ⑩_GREEN account for 67% of a pixel.

Figure 5a shows the proposed pixel layout of 10 × 10 biosensor array. Each pixel is surrounded by a driving electrode and a sensing electrode, the double stacked tower pixel sensor is located in the sensing electrode area. Each pixel sensor in the 10 × 10 array is independent, the driving electrode in each pixel transmit the electrical field, and the transmitted electrical field is read by sensing electrode and readout circuit. There are 100 pixels readout data in a 50 frame/s to show the change of the electrical field due to ion binding in the analyte solution. The readout circuits and system architecture will be introduced in section III. In Figure 5b, the detailed key dimension of the proposed 3D double stacked biosensor are highlighted. Those optimized dimensions in pixel sensor were evaluated through EDA HFSS, the simulated data are shown in Figure 6, Figure 7 and Figure 8. In Figure 6, it shows the investigation to determine the optimization of driving electrode dimension (D) versus sensing electrode (S), and 1st tower width dimension versus 2nd tower width dimension. In Figure 7 and Figure 8, based on the simulation results in Figure 6, we derive the optimization results for dimension D, S, T1 and T2 within a pixel, and then we use FHSS to evaluate the capacitance change for different volume of ratio in single tower structure of Figure 7 and double stacked tower structure in Figure 8. They are studied in the following paragraph.

In Figure 6, we study the maximum capacitance change in relation of sensing electrode width (S) with driving electrode width (D), the 1st tower width (T1) and 2nd stacked tower width (T2). A farther distance from the driving electrode to sensing electrode will cause weaker electrical field, resulting in poor sensitivity, but a short distance magnifies the inherent capacitance, the capacitance change caused by ion binding will be ignored. Therefore, the optimization of distance from driving to sensed electrode is important. Figure 6a shows when width of sensing electrode is increased from 50 μm to 70 μm, the maximum capacitance change occurs at width of driving electrode is 80 μm and width of sensing electrode is 60 μm.

In Figure 6b, we investigate contour fields for double stacked width T1 of 1st tower and width T2 of 2nd stacked tower. Double stacked tower in a pixel sensor increases the volume ratio of a pixel, which will contain more charged groups, resulting the more ion binding in the analyte solution and increasing the higher sensitivity of the biosensor. Figure 6b indicates when the first tower width (T1) is 53 μm, and second tower width (T2) is 25 μm, the maximum capacitance change is observed.

In order to verify the capacitance change caused by ion binding in single tower and double stacked tower, we evaluated the capacitance changes of a pixel with the driving and sensing scheme with the high and low voltage (5 V, 0 V) and driving frequency 50 kHz. Figure 7 shows the contour fields of capacitance change for the single tower condition when the driving and sensing scheme is activated, the unit pixel in the proposed 10 × 10 biosensor is 80 μm pitch. The largest capacitance change is 12 fF, in the hot zone labeled ⑤_RED in the inset of Figure 7, and the second change is labeled ⑥_YELLOW at 9 fF and third change is labeled ⑦_GREEN at 5 fF. The sensitive area including region ⑤_RED, ⑥_YELLOW, ⑦_GREEN in a pixel due to the capacitance change accounts for 36% of a pixel area. The simulation was also performed on double stacked tower structures, as shown in Figure 8, the hot zone of capacitance chance is labeled ⑧_RED at 12 fF, and second change is labeled ⑨_YELLOW at 9fF and third change labeled ⑩_GREEN at 5fF. The sensitive area including ⑧_RED, ⑨_YELLOW, ⑩_GREEN in a double stacked tower pixel account for 67% of a unit pixel area. Compared with Figure 7 and Figure 8, the capacitance change area in unit pixel for single tower structure is less than that of double stacked tower structure. It indicates the high volume ratio of double stacked tower structure makes the capacitance change in a unit pixel increase by 31% comparing to the area of the single tower structure.

### 2.2. System Architecture

#### 2.2.1. System Architecture of Frontend Circuits

**Figure 9:**
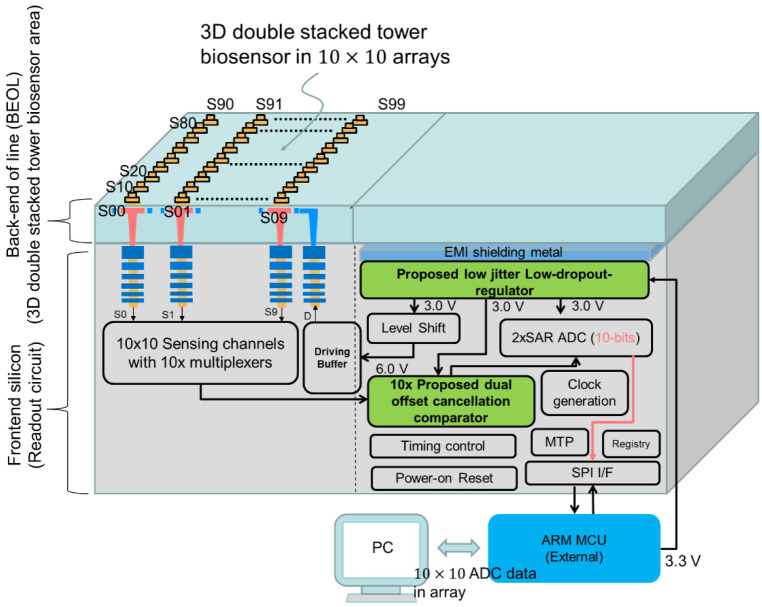
System architecture of readout circuit to read the capacitance change from stacked biosensor in 10 × 10 arrays. The readout circuit includes a proposed low-jitter dropout regulator, and a proposed dual offset cancellation comparator.

Since the capacitance change due to the electrical field change is small, only femto farads, the readout circuit plays an important role in amplifying the capacitance change and sending the correct signal to the subsequent processing unit. Figure 9 illustrate the proposed system architecture. System power is 3.3 V fed into the proposed low jitter low-dropout-regulator (LDO) and regulated to 3.0 V as in-circuit power. Since the sensing capacitance change of the biosensor array varies in femto farad, and is susceptible to power noise from the supply voltage, the power supply rejection ratio (PSRR) in low jitter LDO has to be outstanding. The proposed low jitter LDO circuit is described in sub-section C. The driving scheme is generated by a level shift from 3.0 V in-circuit supply voltage to 6.0 V from a driving buffer, the driving mode is continuouspulse, and send to the driving electrodes in the pixels for 10 × 10 biosensor array. 10 × 10 biosensors sense the capacitance change, the sensing signals from 10 × 10 biosensor array send back to multiplexer circuit, which in turn is read by subsequent dual offset cancellation comparator circuit. The cancellation comparator circuit is designed to offset the mismatch of the threshold voltage in the transistors due to the process variation in the frontend silicon. The offset signal after the cancellation circuit is amplified and sent to a 10-bit analog-to-digital convertor (ADC). The digitized data is compiled into the SPI protocol and sent to external Micro Computing Unit (MCU). The changes in sensed capacitance of 10 × 10 biosensor array were digitized and displayed as an image in 50 frame/second and monitored in computer. All readout circuits shown in Figure 9 are based on the 180 nm bulk CMOS silicon.

#### 2.2.2. Design of Dual Offset Cancellation Circuit

**Figure 10:**
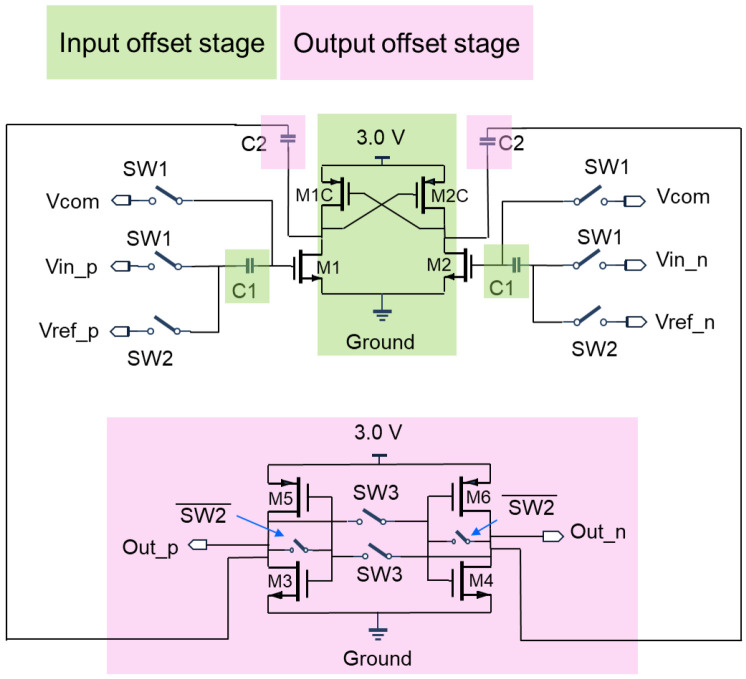
The proposed low offset comparator using dual offset technique. Input stage circuit samples the input offset signal and hold by capacitance C1, output stage circuit read the data from C1 and offset again in C2. A latch circuit in output stage increases the speed of comparison.

**Figure 11:**
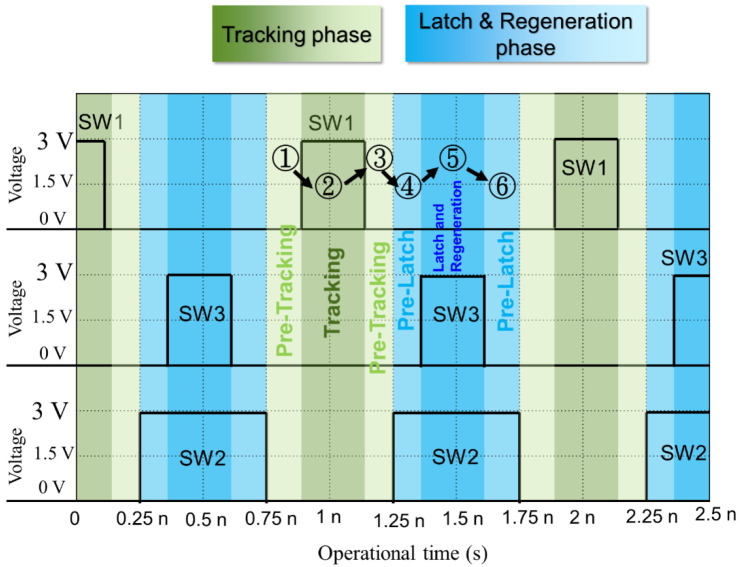
The time sequence of proposed dual offset cancellation comparator.

In Figure 10, the proposed dual offset cancellation comparator includes input stage, the transistors M1, M2, M1C, M2C, and latch and regenerative stage, the transistors M3, M4, M5, M6, and dual offset cancellation stage, the capacitors C1, C2. Transistors M1 and M2 are the differential input loaded with an active cross couple transistor M1C and M2C to accelerate the transient response of transistor M1. When gate terminal in M1 is high and the drain terminal in M1 is turn to low, M2C is turned on immediately and have the drain of M2C turn to high, leading transistor M1C to turn off immediately. When gate terminal in transistor M2 is low and drain in M2 is high, transistor M1C is turned off immediately. In the latch and regenerative stage, the transistor M3 M5, and M4 M6 are serves as back-to-back connected inverters, it is a clock regenerative chain and enable to shorten the delay time in a comparison cycle. In dual offset cancellation stage, the operation sequence for the switch SW1, SW2 and SW3 is illustrated in Figure 11. The input signal with referred offset voltage from Vin_p and Vin_n are sampled and stored in capacitance C1, output offset voltage is stored in capacitor C2. Capacitance C1 is larger than the C2 since larger capacitance reduce the device intrinsic variation, such as offset voltage and transistor mismatch. The input stage transistor M1 M2 can be recognized as a differential amplifier and suppose the gain is large, we have the M2 input as virtual ground, therefore the cancellation techniques for input referred offset in transistor M1 can be obtained as below,

(2) $$V_{out_{p}}=V_{C2}+V_{OS_{M1}}=\frac{C1}{C2}\times V_{in_{p}}+V_{OS_{M1}}$$

*V_OS_M_*_1_ is the offset voltage from transistor *M*1. Since $V_{out\_p}$ = 0, so the input referred offset voltage can be expressed as $V_{OFFSET}=-\left(\frac{C2}{C1}\right)\times V_{OS\_M1}$.

Figure 11 show the operational sequence for the dual offset cancellation scheme. In pre-tracking phase ①, the comparator is in the initial condition. In tracking phase ②, switch SW1 is turned on, capacitance C1 read in the input signal with its offset voltage from Vin_p and Vin_n. When a reference voltage connected to Vcom, the input stage transistor M1 and M2 are biased at Vcom, it operated in the saturation region with transconductance gain. Again the comparator enters pre-tracking phase ③, the input signal voltage with referred offset voltage is stored in capacitance C1. The operation sequence in ① and ③ is in non-overlapping scheme to make sure the outside residue charge not to inject to transistor M1 and M2 channel through parasitic capacitance C_gs_ and C_gd_ in M1 and M2.

In pre-latch phase labeled ④ in Figure 11, the switch SW2 turned on, and SW3 turned off. Reference voltage from Vref_p and Vref_n force bias of capacitance C1 to saturate the transistor M1 and M2, in the meantime, $\bar{\mathrm{SW}2}$ disconnected the drain and gate terminals in transistor M3 M5 and M4 M6, leading the back-to-back inverters chain for M3 M5 and M4 M6 operated in pre-latch stage. The pre-latch phase labeled ④ in Figure 11 prevents the shot voltage (noise) from instant turn-on of SW2, wrongly increasing the charge in C1 and C2. In the latch phase labeled ⑤, the switch SW3 is turned on and force back-to-back inverters M3 M5 and M4 M6 in the latch stage. Input referred offset voltage stored in capacitance C2 are then delivered to output port Out_p and Out_n. At this time, the latch operates as a clock regenerative, thereby increasing the comparison speed. In the phase labeled ⑥, SW3 is turned off again and SW2 still on, the comparator enters again in the pre-latch phase, it completes a comparison cycle.

**Figure 12:**
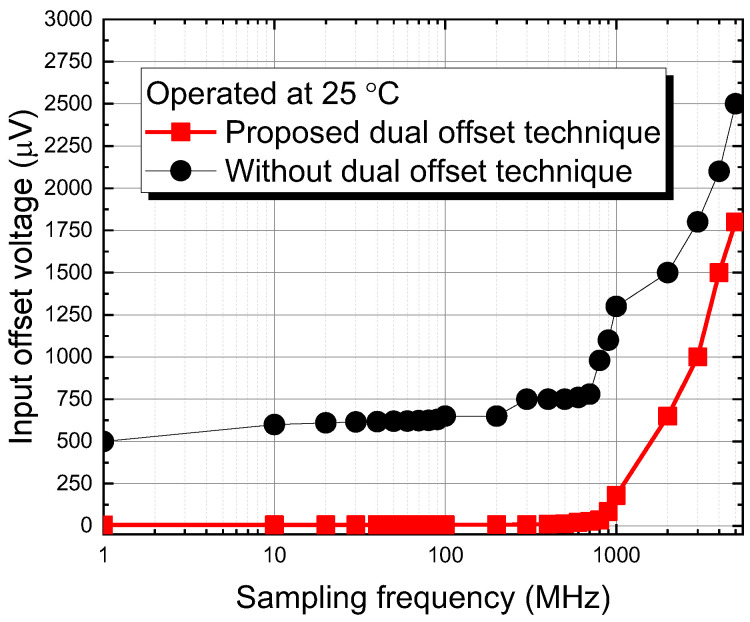
The measured result of the proposed comparator using dual offset cancellation technique.

**Figure 13:**
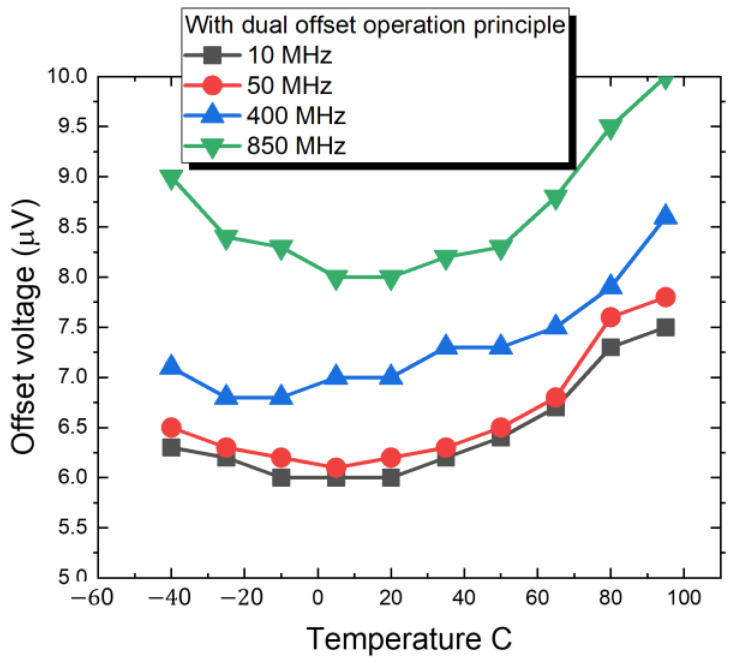
The measured offset voltage related to temperature and sample and hold frequency.

Figure 12 show the measured results for the proposed dual offset cancellation scheme, the low input offset voltage is 8 μV @ 25 °C until 850 MHz, compared to the circuit without dual offset technique, it worse to 750 μV until 850 MHz. In Figure 13, we further to investigate the drift of the offset voltage in dual offset comparator in relation to temperature from −40 °C to 100 °C. In 850 MHz operation frequency, the drift voltage is 9 μV for −40 °C, 8 μV for 20 °C, and 10 μV for 100 °C. The drift with temperature is acceptable. The reason to investigate the temperature effect on the biosensor and readout circuit is due to the chip is often immersed in analyte solution, we always heat the solution to increase the process of ion binding. The smaller the drift voltage in offset, the more accurate and linear the measurement will be.

#### 2.2.3. Design of Low-Jitter Dropout Regulator

**Figure 14:**
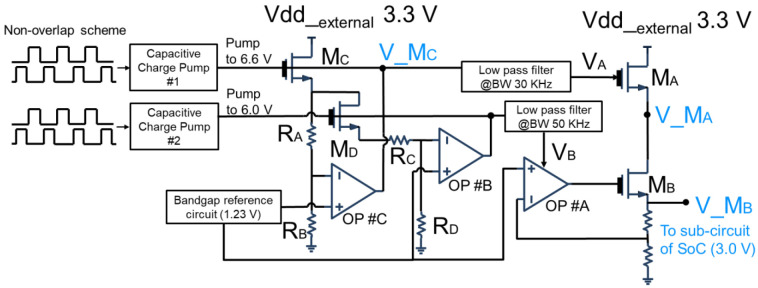
The proposed low-jitter dropout regulator.

Figure 14 shows the circuit scheme of the proposed low-jitter dropout (LDO) regulator. The system supply voltage is 3.3 V externally and we generate a high-power supply reject ratio (PSRR) output 3.0 V as in-circuit voltage for the readout circuit. The proposed low-jitter LDO includes a capacitive charge pump, a non-overlap clock generator, three operation amplifiers and a cascade output stage. The supply voltage with perturbations is fed to N-type transistor M_C_ and M_D_, and M_C_ and M_D_ are the thick oxide gate transistors. Both the gate terminals of transistors M_C_ and M_D_ are supplied by a pair capacitive charge pump. The overdriven voltage 6.6 V and 6.0 V on gate terminal of transistor M_C_ and M_D_ generates high current flowing into the resistors R_A_, R_B_, R_C_ and R_D_, leading to the relatively high dynamic response in amplifiers labeled OP#C and OP#C, which is unaffected by supply power noise.

N-channel transistors M_A_ and M_B_ in the output stage increases higher power supply noise rejection than that of P-type transistors in output stage [19]. Transistor M_A_ and M_B_ are configurated in common gate, N-type transistors provides a higher impedance, therefore a better isolation to supply voltage perturbations is obtained for N-type transistors. Though the body terminal in transistor M_A_ picked up to ground will cause the body effect, leading several millivolts increase of threshold voltage, we require boosting the output voltage V_M_B_ in the output stage by several hundred millivolts to make sure the voltage headroom for subsequence analog circuits. The voltage V_A_ and V_B_ from capacitive charge pumps are to bias the stacked N-type transistor M_A_ and M_B_. Since V_A_ and V_B_ are boosted by the capacitive charge pump circuits that may contain high frequency noise, a pair RC lowpass filters are subsequently added. Meanwhiles, the low frequency noise spectra lying withing the operational amplifier OP#A bassband is attenuated because of the feedback being utilized in the amplifier.

We use a first order small signal model to evaluate the transfer function $\frac{V\_M_{B}\left(s\right)}{V_{external}\left(s\right)}$ for low-jitter LDO in Figure 14, which means a measurement of the power supply noise rejection with respect to the supply voltage externally,

(3) $$\frac{V\_M_{B}\left(s\right)}{V_{external}\left(s\right)}≅{\left(A_{OP\#A}\right)}^{-1}\times \left(\frac{g_{ds_{MA}}}{g_{m_{MA}{}_{\;}-}g_{mb_{MA}}}\right)\times \left(\frac{g_{ds_{MB}}}{g_{m_{MB}{}_{\;}-}g_{mb_{MB}}}\right)\;\;\times \;\frac{\left(1+\frac{s}{P_{A\_OP\text{\#}A}}\right)}{\left(1+\frac{s}{P_{1}}\right)\left(1+\frac{s}{P_{2}}\right)\left(1+\frac{s}{Z_{A\_OP\text{\#}A}}\right)}$$

where

(4) $$P_{1}=\frac{g_{m\_MA_{\;}-}g_{mb\_MA}}{C_{Loading}}$$

(5) $$P_{2}=\frac{g_{m\_MB_{\;}-}g_{mb\_MB}}{C_{Loading}}$$

$g_{ds}\;$__*MA*_, $g_{m\_MA}$ and $g_{mb\_MA}$ represent the small signal drain-source conductance, forward transconductance and bulk transconductance of transistor $M_{A}$; $g_{ds\_MB}$, $g_{m\_MB}$ and $g_{mb\_MB}$ are for transistor of $M_{B}$; $A_{OP\#A}$ is the low frequency gain of operation amplifier OP#A; $P_{A\_OP\#A}$ and$Z_{A\_OP\#A}$ are the dominant pole and zero of operational amplifier OP#A.

**Figure 15:**
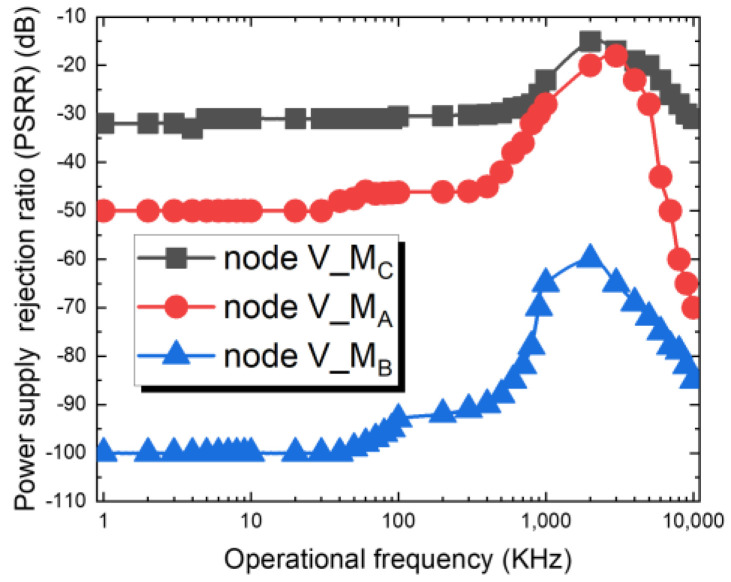
The frequency response of the supply noise in node V_M_C_, V_M_A_, and V_M_B_. The spectrum has bandpass characteristics, and both the low frequency and high frequency noise components are attenuated. The PSRR equation is H(s) = V_M_A/B/C_(s)/V_dd_ (s), the frequency domain the locations of concomitant poles and zeros, and the resultant contribution of power supply noise to the overall noise properties of the loop.

Figure 15 shows the results of power supply rejection ratio (PSRR) frequency response for the nodes of V_M_C_, V_N_A_ and V_M_B_ denoted in Figure 14. In the output stage node V_M_B_, the PSRR is obtained −92dB @ 100 kHz, −65dB @ 1 MHZ.

**Figure 16:**
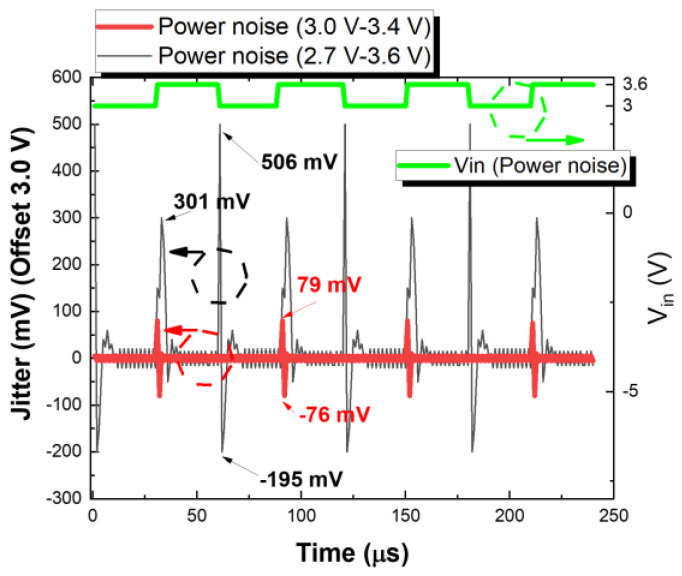
The measurement of output jitter from node V_M_B_ of proposed low jitter low dropout regulator. The measured jitter is offset by 3.0 V. The jitters are investigated by power noise from 3.0 V to 3.4 V, and 2.7 V to 3.6 V.

Figure 16 depicts the time domain jitter measurements for the node V_M_B_ in the output stage, it is offset to 3.0 V. A 20 kHz supply voltage noise with (3.0 V to 3.4 V) and (2.7 V to 3.6 V) is fed to power pad of LDO, the measured jitter range of output voltage from node V_M_B_ are (79 mV, −76 mV) and (301 mV, 195 mV).

## 3. Results

**Figure 17:**
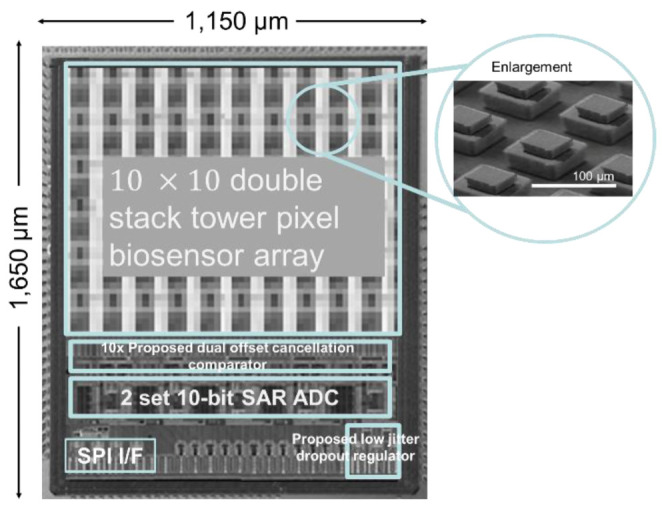
The micrograph of proposed biosensor in array and integrated readout circuit on the same silicon substrate. The 3D MEMS-based double stacked tower biosensor is shown.

Figure 17 illustrates the SoC die microphotograph of the proposed double stacked tower biosensors and readout circuit, the microscopic view of the biosensor array is shown in the inset of the Figure 17. The proposed dual offset cancellation readout circuit is placed next closely to 10 × 10 layout sensor in order to sense signal with less noise coupling from the environment. Next are 2 set successive approximative recession (SAR) 10-bit analog to digital convertor (ADC), it digitizes the biosensor analog signal to the following serial peripheral interface (SPI) circuit. The proposed low-jitter low dropout regulator is located at corner of chip with the shortest distance from power pad pin to reduce current times resistant effect (IR effect). The chip area is 1150 μm × 1650 μm and is fabricated using 180 nm CMOS bulk process with MEMS back-end of line process.

**Figure 18:**
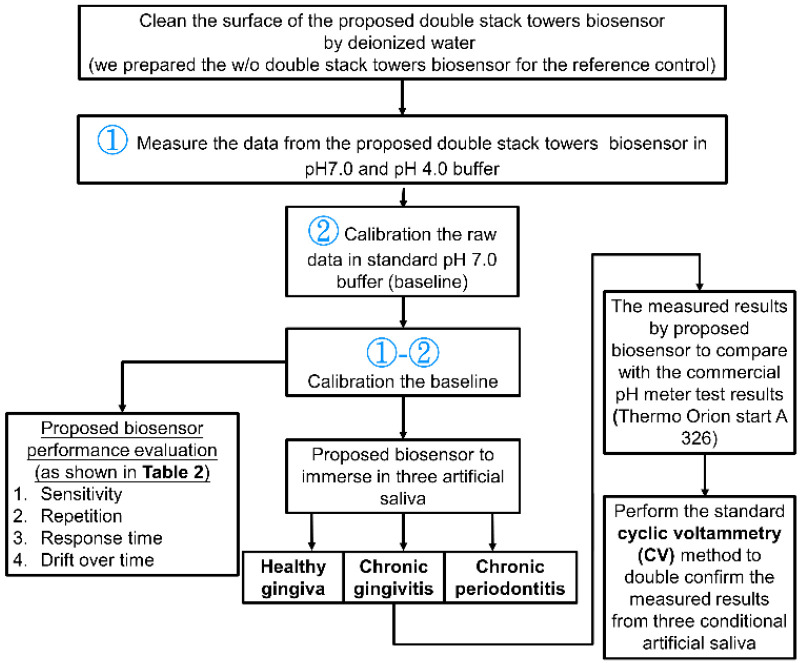
The calibration and measurement procedure for three artificial saliva, they are healthy gingiva, chronic gingivitis and chronic periodontitis, respectively. The measured result of proposed biosensor was also double confirmed by a commercial pH meter and cyclic voltammetry (CV) method to demonstrate the consistency of the results.

Figure 18 shows the measurement and calibration flow to evaluate the proposed biosensor and readout circuit. The proposed biosensor was immersed in the pH 7.0 and pH 4.0 solution, the readout data at pH 4.0 calibrated the data at pH 7.0, as pH 7.0 was used as baseline. We investigate the characteristics of the proposed biosensor in view of sensitivity, repetition, response time and drift over the time, the performance is summarized in Table 2. After characterizing the biosensor performance, we immersed biosensor to artificial saliva from the condition of healthy gingiva, chronic gingivitis, and chronic periodontitis. The measured data were compared with the commercial pH meter Thermo Orion start A 326, and we also performed the cyclic voltammetry (CV) method to extract capacitances of saliva under those conditions.

**Figure 19:**
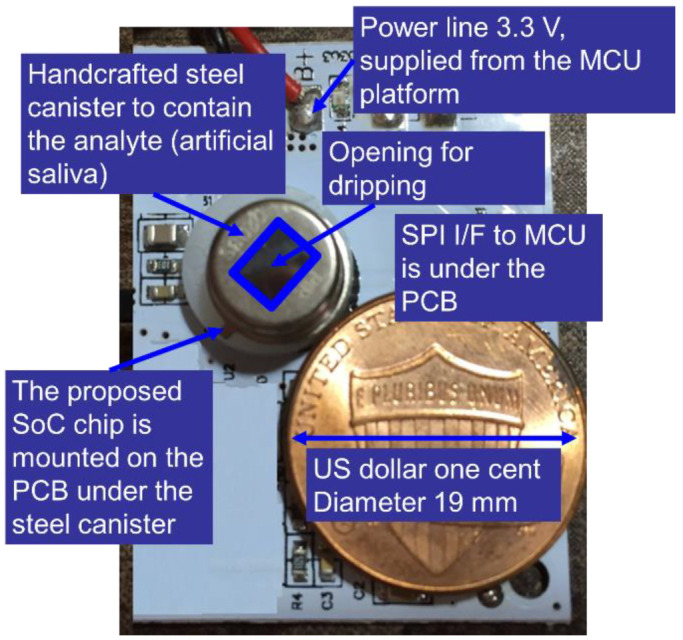
The system view of the proposed biosensor with integrated readout circuit on the FR4 print-circuit-board.

**Figure 20:**
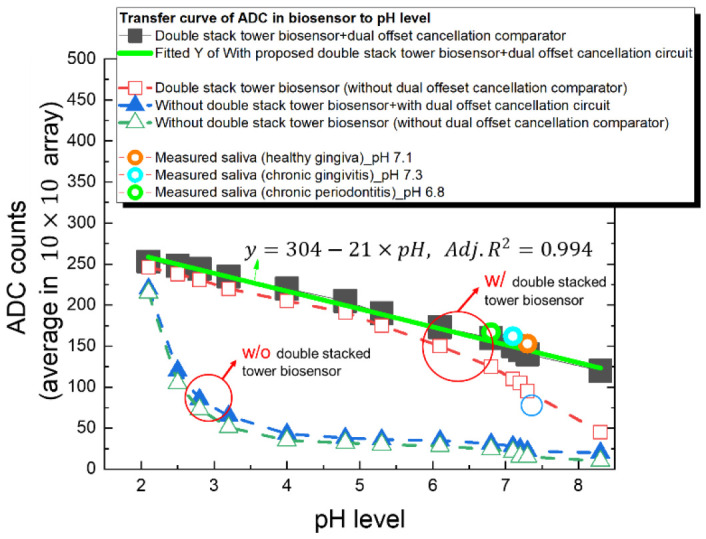
The transfer curves of ADC read-counts versus pH level in proposed biosensor. The fitted equation (green line in Figure) of the proposed biosensor with dual offset cancellation comparator shows a wide linear range from pH 2.0 to pH 8.3. Without double stacked tower biosensor, the overall linearity is divided into two parts, with steeper sensitivity at pH 2.0–3, and less sensitivity at pH 3.0–8.3, making it difficult to distinguish the chronic disease gingiva saliva at pH 6.8–7.3.

Figure 19 illustrates the system level photograph of biosensor and readout circuit. The chip is mounted on FR4 type print-circuit-board. A handcrafted steel canister with an opening at top covers the chip, and the analyte drips from opening of the canister. Figure 20 shows the ADC readout out results against the various pH solutions and conditional saliva. The measured results with the proposed double stacked tower biosensor structure show a good linearity from pH 2.0 to pH 8.3, especially the measured data of stacked tower biosensor with double offset cancellation circuit show a highly linearity than that without double offset cancellation circuit. The one order fitting parameter is shown in inset of Figure 20, the square error R^2^ is 0.994. We observe the double stack biosensor without using offset cancellation comparator shows a poor linearity in the range of pH 6 to pH 8.

Comparing the measured result without double stacked tower biosensor, the linearity range versus pH level is poor. In the range of pH 2.0 to pH 3.0, the electrical field in solution is absorbed by the accumulated ion, so the slope of ADC counts versus pH 2.0 to pH 3.0 level is steeper, and the ADC counts shows a strong sensitivity; however, when solution pH is in range pH 3.0 to pH 8.3, the ion in solution reduced, resulting in a decrease in ion binding activity, and the sensitivity for ADC against pH level becomes insensitive. Although the results without double stacked tower biosensor can still detect the electrical field change as a function of pH level, it is difficult to accurately predict the pH of analyte when the electrical field change varies very little, such as range from pH 5.0 to pH 8.0.

After characterizing the proposed biosensor, we further investigate three artificial saliva pH value for healthy gingiva, chronic gingivitis and periodontitis. In Figure 20, the pH of saliva for three cases are matched to the fitted curve of the ADC counts. We observe the double stacked biosensor without using offset cancellation comparator shows a poor linearity in the range of pH 6 to pH 8.

**Figure 21:**
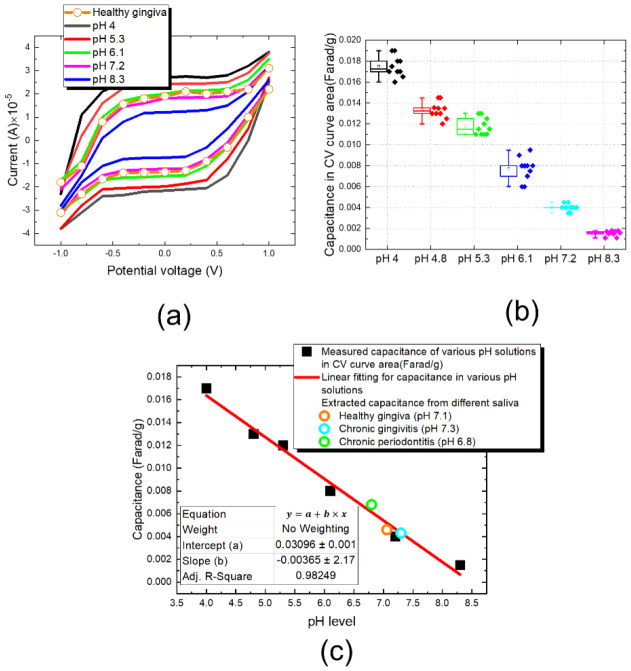
Investigation of various pH solutions and chronic disease gingiva saliva studied by using cyclic voltammetry (CV) method. (**a**) The relationship between measurement current and scan voltage, and (**b**) the capacitance integration under the area of CV curve in (**a**,**c**) the fitted curve (red line) covers the measured capacitances of three artificial disease saliva.

In order to further evaluate the confidence level of the measurement data in Figure 20, we utilize traditional cyclic- voltammetry (CV) methodology to extract capacitance of pH solution and saliva. In Figure 21a, the measured current for various pH solution in cyclic potential voltage from −1.0 V to 1.0 V at a scan rate of 0.01 V/s is shown. Afterwards, capacitances for different pH solutions are calculated based on the integration of CV curve area in Figure 21a. We repeat 10 CV measurements for each pH solution, and the measured capacitances are shown in Figure 21b. Finally, we plot the capacitances integrated from CV curve in Figure 21a against pH level and is shown in Figure 21c. In Figure 21c, the fitted equation is highly linearity from pH 4.0 to pH 8.3 with root square error 0.982. Meanwhiles, the capacitances of three conditional of artificial saliva, periodontitis (pH 6.8), healthy gingiva (pH 7.1) and chronic gingivitis (pH 7.3) measured by using the cyclic-voltammetry methodology are proceeded, the results are linear fitted to equation.

Table 2 summarize the proposed 3D double stacked tower biosensor array with dual offset cancellation comparator and low-jitter LDO. This work features with a linearity over a wide range of pH 2.0 to pH 8.3, sensitivity of −21 ADC counts/pH (or 212 mV/pH), response time of 5 s, repetition of 98.9%, drift over time of 0.5 mV. The analyte target is chronic gingiva disease saliva. Our proposed biosensor exhibits good resolution and good linearity in the chronic periodontal disease saliva range from pH 6.8 to pH 7.3.

## 4. Conclusions

The prevalence of periodontal disease is as high as 50% in the world, and about 47% of them have serious problems. Except for regular inspection in the hospital, there is currently no simple medical device as a Point of Care that allows patients to perform immediate pre-periodontal disease testing at home. In this paper, we design and implement a pH sensing semiconductor device by proposed a MEMS based double stacked tower biosensor and readout circuit.

In this work, we demonstrated a 3D stacked tower pixel biosensor array with wide-range linearity from pH 2.0 to pH 8.3 to diagnose chronic periodontal disease. Based on the site-binding theory, we increase the volume ratio of biosensor by proposing 3D double stacked tower pixel structure to enhance the charge group concentration. The stacked tower biosensor array is fabricated by MEMS back-end of line by the CMOS bulk process.

We further investigate the variation of the stacked tower electrical field change with respect to tower geometry and the associated driving and sensing electrode width in a pixel based on a modified Cole-Cole model. Based on the model simulation, the sensitivity of double stacked tower within a unit pixel is 31% higher than that of single stacked tower.

The 3D double stacked tower biosensor array is highly integrated with readout circuit in the frontend 180 nm CMOS bulk process as a system-on-chip. The proposed dual offset cancellation circuit in readout circuit has a low input offset voltage of 8 μV @ 25 °C until 850 MHz. Meanwhiles, in order to isolate external power noise, a low-jitter dropout regulator is implemented in readout circuit to provide a stable 3.0 V voltage for SOC’s sub-circuits. When power line is perturbated with 400 mV at 20 kHz noise, the measurement of jitter in highest and lowest is (79 mV, −76 mV), and PSRR is −92 dB at 100 kHz and −65 dB at 1 MHZ.

Based on our 3D stacked tower biosensor and excellent readout circuit with a dual cancellation offset comparator and a low-jitter LDO circuit, the biosensor exhibits wide-range linearity from pH 2.0 to pH 8.3, and high sensitivity of −21 ADC counts/pH (or 212 mV/pH), response time of 5 s, repetition of 98.9%, and drift over time of 0.5 mV. The proposed 3D biosensor SoC can discriminate between healthy gingiva pH 7.1, chronic gingivitis pH 7.3, and chronic periodontitis pH 6.8, and the measurements has been successful verified with traditional cyclic-voltammetry (CV) method.

In the future, the proposed 3D double stack tower biosensor and readout circuit in this work can be further integrated with internet-of-thing (IoT) device and NFC for power data-transmission and data-communication for continuous pH sensing to facilitate the gingiva health care at home.

## Figures and Tables

**Table 1:** The parameter lists of modified Cole-Cole model and extraction results in solution of pH 7.2 and pH 4.0. α is a dispersion coefficient evaluating the relaxation time constants in an analyte.

	Analyte	Solution pH 7.2	Solution pH 4.0
Parameters			
RP_target		18.2 KΩ	13.8 KΩ
R_target		2.8 KΩ	2.1 KΩ
C_target		$2.94\times 10^{-9}\;$ F	$21.2\times 10^{-9}\;$ F
α		0.81	0.75

**Table 2:** Summary of the performance of the proposed double stacked tower sensor array with readout circuit and comparison with prior works.

	Functions	Principle	Linearity Range	Sensitivity	Response Time in Second	Repetition	Drift over Time	Analyte Target	Functional Type
Prior Works									
This work w/3D double stacked tower array + w/dual cancellation comparator		Electrical field	Wide pH 2.0–8.3	−21 ADC count/pH (212 mV/pH)	5 s	98.9%	0.5 mV	Chronic gingiva disease saliva	10×10array pH Image (50-frame/s)
This work w/o 3D double stacked tower array+ w/o dual cancellation comparator			Narrow Range 1: pH: 2–3 Range 2: pH: 3–8.3	−6 ADC count/pH	7 s	96%	0.5 mV	Chronic gingiva disease saliva	
[20] Y-2017		Electrical field	Narrow Range 1: pH: 4–6 Range 2: pH: 6–10	187 nA/pH	6 s	90%	2nA([11] IDE NO:1–5)	Alkali and hydroxyl buffer	Analog current
[21] Y-2018		Electrical field	Narrow pH: 5–8	0.64 μF/pH	13 s	92%	7.1 mV	Glucose, Na^+^. K^+^	Analog voltage
[10] Y-2020		Transistor channel current	Narrow pH: 6–7.6	140 mV/pH	20 s	93%	3.2 mV	Standard buffer	Analog voltage
[11] Y-2019		Transistor channel current	Wide pH: 7–12	49 mV/pH	24 s	91%	4.2 mV	Standard buffer	Analog voltage
[22] Y-2017		Transistor channel current	Narrow Range 1: pH: 4–6 Range 2: pH: 6–10	130 mV/pH	12 s	92%	6.2 mV	Standard buffer	Analog voltage

## Data Availability

Not applicable.
